# Responses to salinity in the littoral earthworm genus *Pontodrilus*

**DOI:** 10.1038/s41598-022-26099-w

**Published:** 2022-12-24

**Authors:** Teerapong Seesamut, Beewah Ng, Chirasak Sutcharit, Ratmanee Chanabun, Somsak Panha

**Affiliations:** 1 Animal Systematics Research Unit, Department of Biology, Faculty of Science, Chulalongkorn University, 254 Phayathai Road, Pathumwan, Bangkok, 10330 Thailand; 2 Freecap Resource Sdn Bhd, Lot T-5, Lumut Port Industrial Park, KG Acheh Mukim Lumut, 32000 Sitiawan, Perak Malaysia; 3 Program in Animal Science, Faculty of Agricultural Technology, Sakon Nakhon Rajabhat University, Sakon Nakhon, 47000 Thailand; 4 Academy of Science, The Royal Society of Thailand, Bangkok, 10300 Thailand

**Keywords:** Ecology, Zoology

## Abstract

The cosmopolitan littoral earthworm *Pontodrilus litoralis* is distributed in tropical and sub-tropical coastal habitats, whereas *P. longissimus* is reported only in the Thai-Malay coastal line. In the present study, we examined the difference in salinity effect on the survival rate, wet weight (hereafter weight) change, behaviour, and osmolality of these two *Pontodrilus* species. A 28 d exposure to varying salinity concentration (0–50 ppt) revealed that *P. litoralis* is able to survive over a wide salinity range than *P.* longissimus, with the latter species exhibiting a low survival rate over the same salinity range. During short-term exposure (0–96 h) to a salinity of less than 30 ppt, *P. litoralis* exhibited weight gain and this was significant in the first 12 h of exposure. However, *P. longissimus* gained weight when exposed to salinity at under 10 ppt in the first 72 h of exposure. The two species of *Pontodrilus* behaved differently when exposed to different salinities. The coelomic fluid osmolarity of *Pontodrilus* was related to the exposure medium and was mostly maintained as hyperosmotic to the external medium over the range of salinities tested. This study shows how two different species of the littoral earthworm genus *Pontodrilus* respond to a change in salinity, which may explain their dispersal pattern and shape their distribution pattern throughout Southeast Asia.

## Introduction

Earthworms are well recognized as soil engineers that significantly affect soil ecosystems by enhancing the soil macro and micronutrients as well as the soil structure^1–3^. Amongst the reported megascolid terrestrial earthworm, *Pontodrilus* is the only genus that is found globally distributed on sandy beaches and coastal areas. At present, two species of the littoral earthworm genus *Pontodrilus* have been recorded from the seashore of Thailand and Peninsular Malaysia namely *P. litoralis* (Grube, 1855) and *P. longissimus* Seesamut and Panha, 2018. Habitat preferences for *P. litoralis* and *P. longissimus* are obviously different with *P. litoralis* being widely distributed throughout tropical and sub-tropical sandy beaches around the world, while *P. longissimus* is only found at specific localities. Moreover, *P. litoralis* can be found in a wide range of habitats, such as sandy beaches, estuaries, mangrove swamps, and the lakes with a very low salinity^4–7^, and different types of soil texture. In contrast, *P. longissimus* can only be found in fine and muddy sand at the ecotone between marine and terrestrial-freshwater aquatic linked habitats^6,8^.

Variation in salinity is a major environmental major influence on the distribution of organisms in estuaries^9–11^. Previous studies reported that increments in the salinity had harmful effects on the growth, mortality, and reproduction of earthworms, while sodium chloride shows negative effects on the earthworm’s life cycle^12,13^. As mentioned above, the littoral earthworm genus *Pontodrilus* are euryhaline and are often exposed to salinity fluctuations that may range between 1 and 33 ppt^6,7^. In addition, the specific euryhalinity character of *Pontodrilus* has been reported in some detail^14,15^, and is believed to be an important tool for the littoral earthworm to regulate the osmotic concentration of their body fluid in order to survive and adapt to that extreme environment.

Earthworms in general can tolerate limited salinity, but some earthworms, like *Eisenia fetida*^16^, are highly salt-tolerant. In this research, the salinity tolerance of *P. litoralis* and *P. longissimus*, which both occur in saline habitats but in different microhabitats^6,17^, was evaluated based upon the following. To verify the distribution pattern of *P. litoralis* and *P. longissimus*, it is necessary to further identify the main driving factor behind this habitat selection process. The present study was, therefore, dedicated to identifying their salinity preferences in determining the successful adaptation of these two *Pontodrilus* species with respect to their recorded habitat. Experimentally, we observed the survival rate, weight change, behavior, and osmolality of *P. litoralis* and *P. longissimus* when subjected to different salinity concentrations over both a short- and a relatively long-term exposure (0–96 h and 28 d, respectively). We evaluated two hypotheses (i) a higher salinity will negatively affect the survival and weight of *Pontodrilus*, and (ii) that, since *P. litoralis* is a cosmopolitan earthworm that occurs naturally in the sand surface areas where sharp salinity gradients are frequent along with a high salinity exposure, it will be able to survive wider salinity ranges and higher salinity concentration than *P. longissimus*, a species that inhabits a deeper level in the sand and preferentially occurs in estuaries.

## Results

### Field sampling and observation

**Figure 1:**
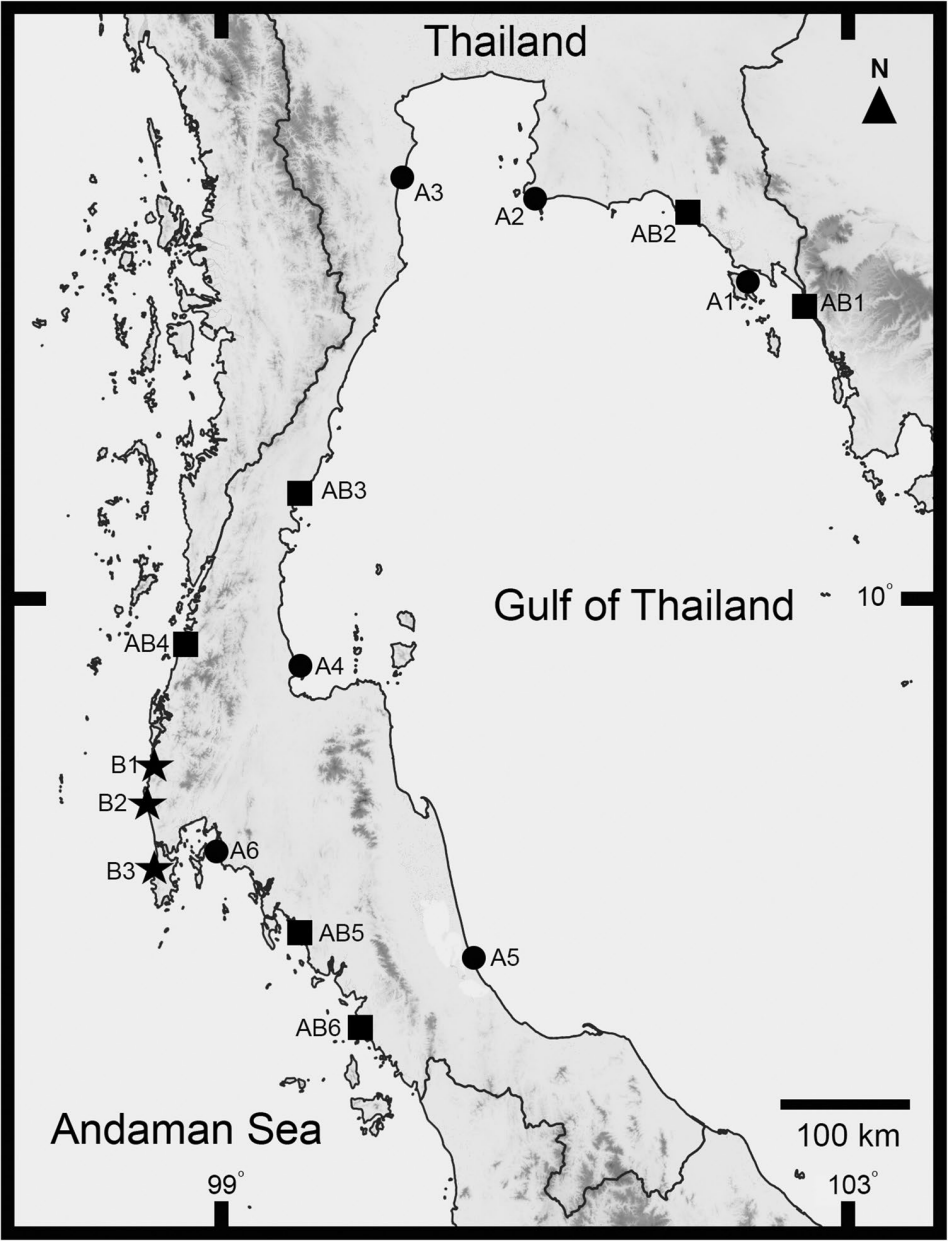
Map of the sampling locations where *Pontodrilus* species were found in this study. Circle and star symbols represent localities for *P. litoralis* and *P. longissimus,* respectively, while square symbols are the localities where both *Pontodrilus* species were found. (Abbr. are mentioned in Supplementary Table 1).

A total of 15 habitats of *Pontodrilus* were observed, and the salinity level was recorded in the field (Fig. 1, Supplementary Table 1, Supplementary Fig. 1). *Pontodrilus litoralis* was found in various habitat types for example under trash or leaf litter on the sandy beach, in mangrove swamp, sanitary sewer emptying to a sandy beach, and estuaries. These sites included both undisturbed and disturbed areas. However, *P. longissimus* mostly occurred in estuaries. We observed six localities where *P. litoralis* was found co-existing with *P. longissimus* in estuary habitats. At these locations, *P. litoralis* was found under debris or trash in the topsoil while *P. longissimus* was found deeper than *P. litoralis*; at a depth of 10–50 cm. The highest salinity recorded in a *P. litoralis* habitat was 33 ppt, whereas it was 21 ppt for sites with *P. longissimus*.

### Long-term (28 d) exposure to different salinity concentrations

**Figure 2:**
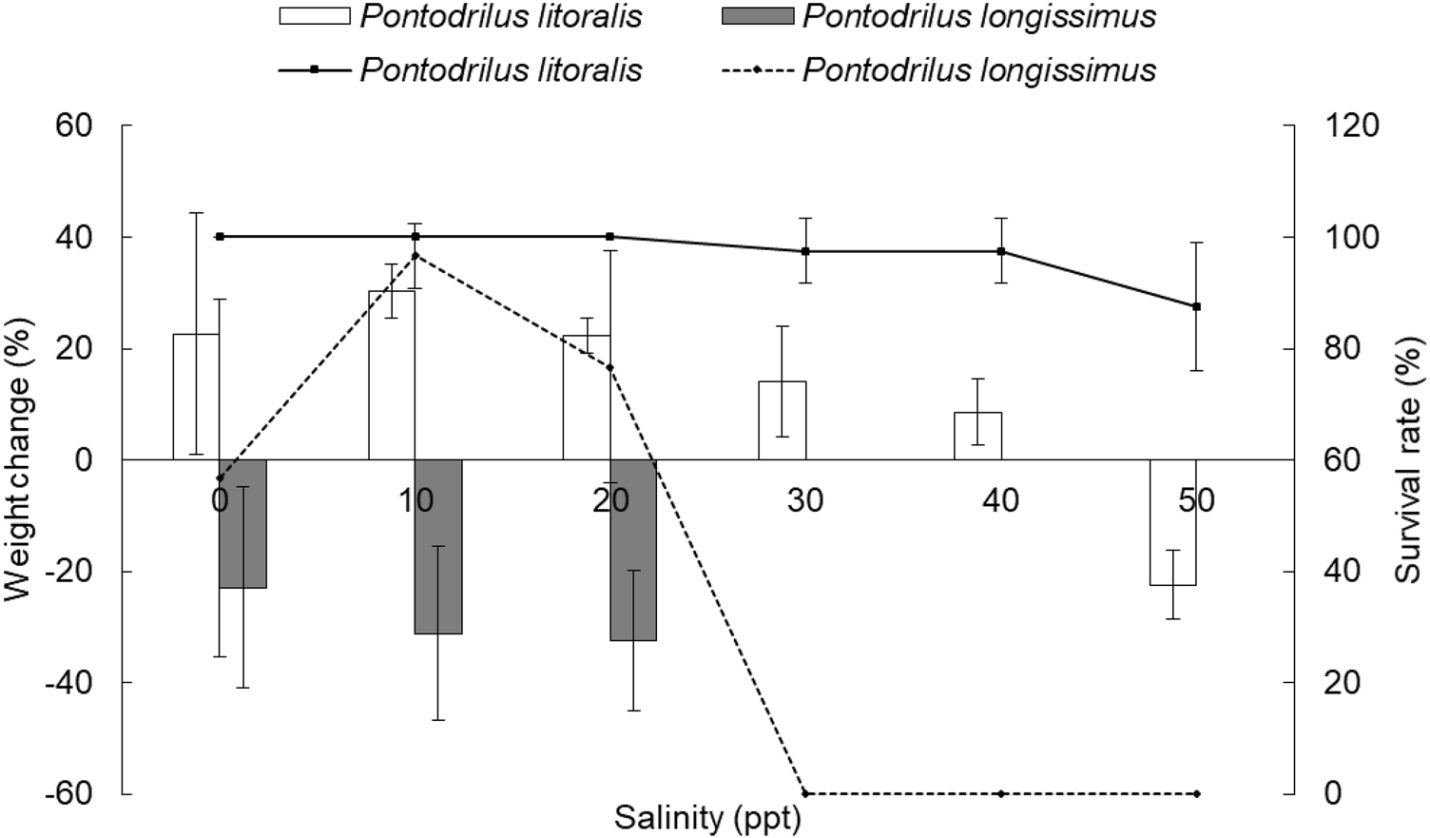
*Pontodrilus litoralis* and *P. longissimus* after a 28-day exposure to different salinity concentrations. The percentage survival rates are shown as lines while the changes in the fresh weight are shown as bars. Data are shown as the mean ± SD, derived from four replications.

Data on the survival rate and weight change of littoral earthworms exposed for 28 d to different concentrations of salinity are presented in Fig. 2. *Pontodrilus litoralis* was able to survive a wide range of salinities (0–50 ppt) with the highest survival rate observed at ≤ 30 ppt. The survival rate then decreased as the salinity increased above 30 ppt, falling to 97.5% and 87.5% at 40ppt and 50 ppt, respectively. In contrast, the survival of *P. longissimus* was more strongly affected by changes in salinity with the highest survival rate recorded at 10ppt (ca. 97%), being lower at 0 ppt (ca. 56%), and decreasing at salinities above 10 ppt to ca. 76% at 20 ppt and 0% at 30–50 ppt. Relatively, *P. litoralis* have a higher survival rate than *P. longissimus* and is better adapted for survival over a wider salinity range.

The weight change of *P. litoralis* was significantly affected by the salinity of the test medium, an increment in weight was observed when *P. litoralis* was exposed to a salinity of less than 40 ppt (highest increase as the salinity increased above 10 ppt to 40 ppt), but with a marked weight loss at 50 ppt. In contrast, *P. longissimus* exhibited a high weight loss at a low salinity of 0–20 ppt and was unable to survive at salinities above 20 ppt. These results clearly indicated that *P. litoralis* is better adapted than *P. longissimus* to changes in salinity, which would likely account for its wide distribution in different environments.

### Short-term (0–96 h) exposure to different salinity concentrations

**Figure 3:**
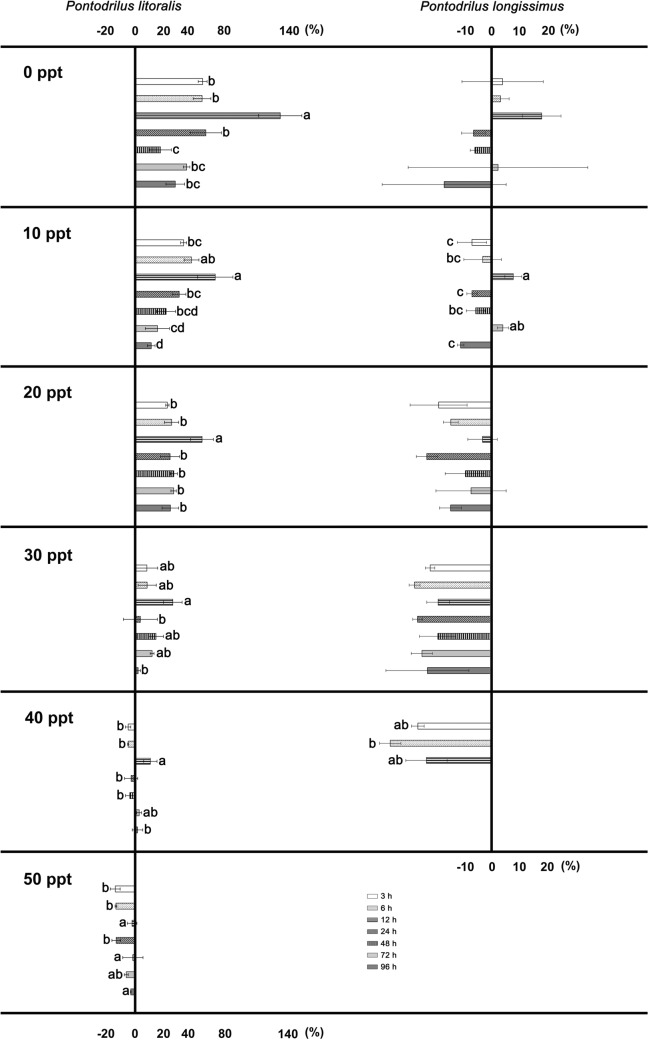
Weight change (%) of adult *P. litoralis* and *P. longissimus* after 3, 6, 12, 24, 48 h, 72 h and 96 h of exposure to different salinity concentrations. Data are shown as the mean ± SD, derived from three replications. Means with a different letter are significantly different (ANOVA: Tukey’s test; P < 0.05).

*Pontodrilus litoralis* exhibited the same weight gain pattern when exposed to a salinity of less than 30 ppt, where a significant weight gain was clearly observed over the first 12 h of exposure (Fig. 3) followed by a sharp decrease in the weight gain and ending in a constant weight after nearly 96 h. A marked weight loss was observed when *P. litoralis* was exposed to a salinity of 40 and 50 ppt. For *P. longissimus*, there was an increment in weight over the first 72 h of exposure to a salinity of 0 ppt and 10 ppt, but a prolonged exposure (96 h) resulted in a significant weight loss*.*

**Figure 4:**
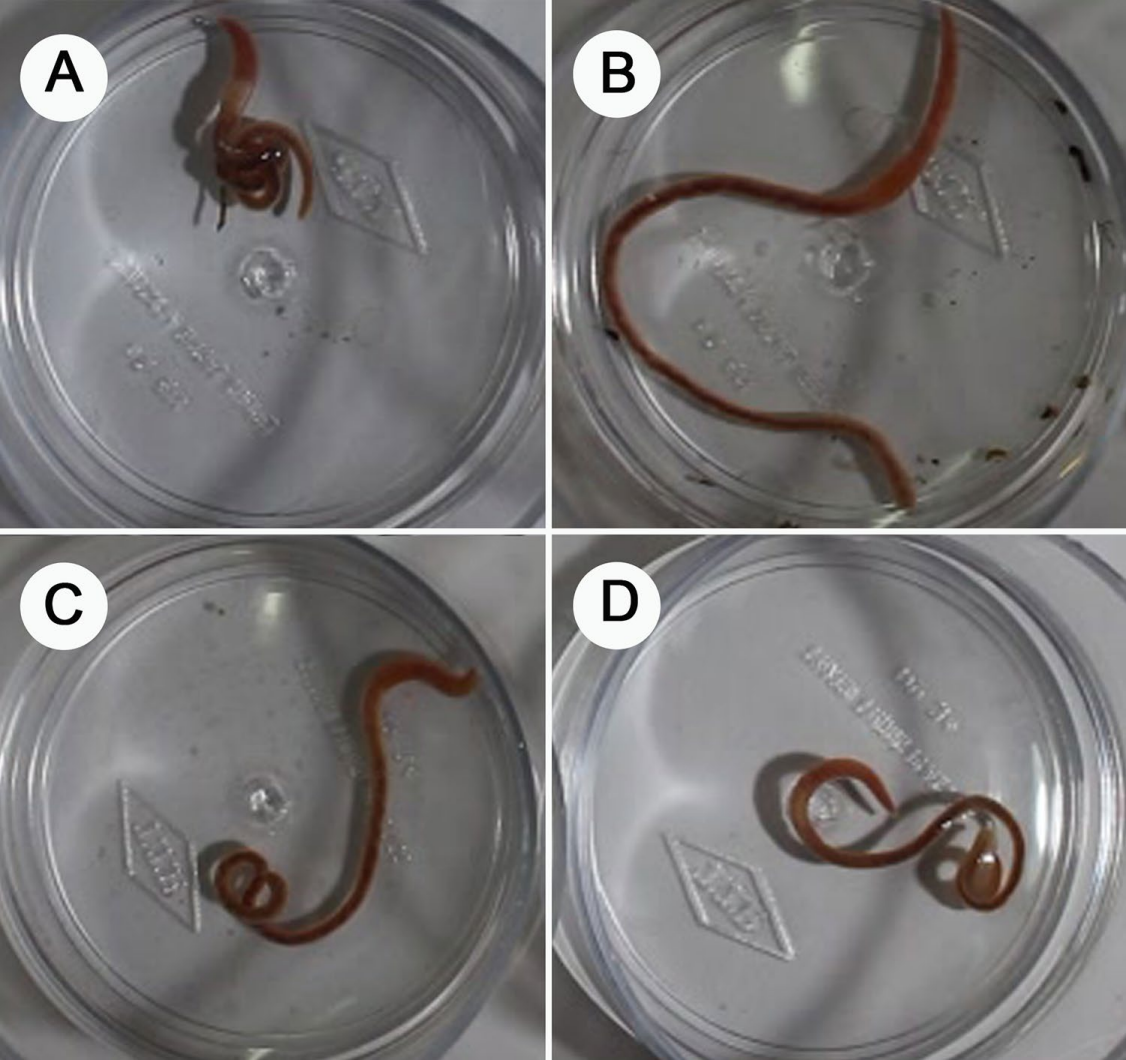
Photographs showing *Pontodrilus* behavior when exposed to different salinities and time: (**A**) coiled and knotted [10 ppt after 3 h of exposure], (**B**) stretching [10 ppt after 48 h of exposure], (**C**) posterior end turns reddish [40 ppt after 6 h of exposure], (**D**) thinner in size at the posterior end [50 ppt after 24 h of exposure].

*Pontodrilus litoralis* and *P*. *longissimus* behaved differently when exposed to different salinity levels; not all individual presented the same behavior patterns initially but towards the end of the exposure period (96 h), all of them showed similar patterns in the introduced test medium. At a salinity of 0–30 ppt, *P. litoralis* immediately coiled and knotted (Fig. 4A) its posterior end. This response was maintained for up to 24 h of exposure and then they started to relax and loosen the knot by fully stretching their body (Fig. 4B) and maintained a calm state with slow movement until the end of the experiment. Increasing the medium’s salinity resulted in an increased body movement in *P. litoralis* with the most active was recorded at a salinity of 30 ppt. At 40 ppt, *P. litoralis* started to loosen its knot and stretch its body after 6 h of exposure, and the posterior end turned reddish (Fig. 4C), but they still moved a moderate amount until the end of the experiment. At 50 ppt, *P. litoralis* was inactive and became thinner at the posterior end (Fig. 4D). Besides these behavioral changes, the size changed significantly when *P. litoralis* was exposed to a high or a low salinity (Supplementary Fig. 2). In contrast, *P*. *longissimus* reacted differently compared to *P. litoralis*. At a salinity of 0–20 ppt, slow movement and coiled at posterior end was observed in *P. longissimus.* After 12 h of exposure, they started to relax and loosen the knot by fully stretching their body until the end of the experiment. At 30 ppt, *P. longissimus* coiled and knotted its posterior end from the beginning of exposure until towards 96 h of exposure. At high salinities (40–50 ppt), *P. logissimus* immediately coiled and knotted its body with rapid movement, after 3 h of exposure *P. longissimus* was inactive and the whole body of the earthworm turned reddish and became thinner with 100% mortality was observed in all replicates of treatment.

### Osmolality of the coelomic fluid

**Figure 5:**
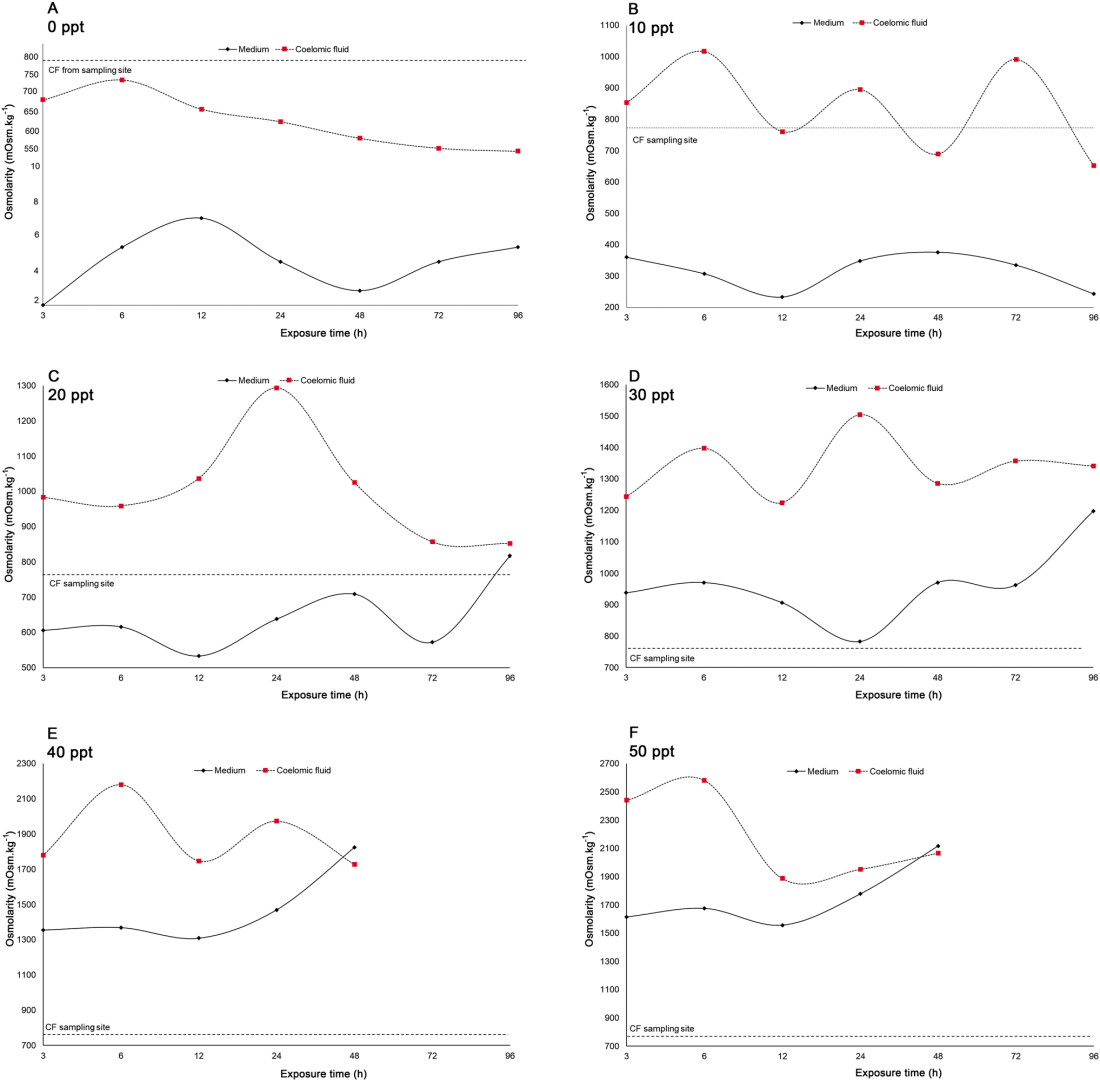
The coelomic fluid osmolarity of *P. litoralis* after exposure to different salinity concentrations of: (**A**) 0 ppt [different scale of Y axis was used to plot the graph], (**B**) 10 ppt, (**C**) 20 ppt, (**D**) 30 ppt, (**E**) (40) ppt, and (**F**) 50 ppt. Horizontal constant dash lines represent field values.

**Figure 6:**
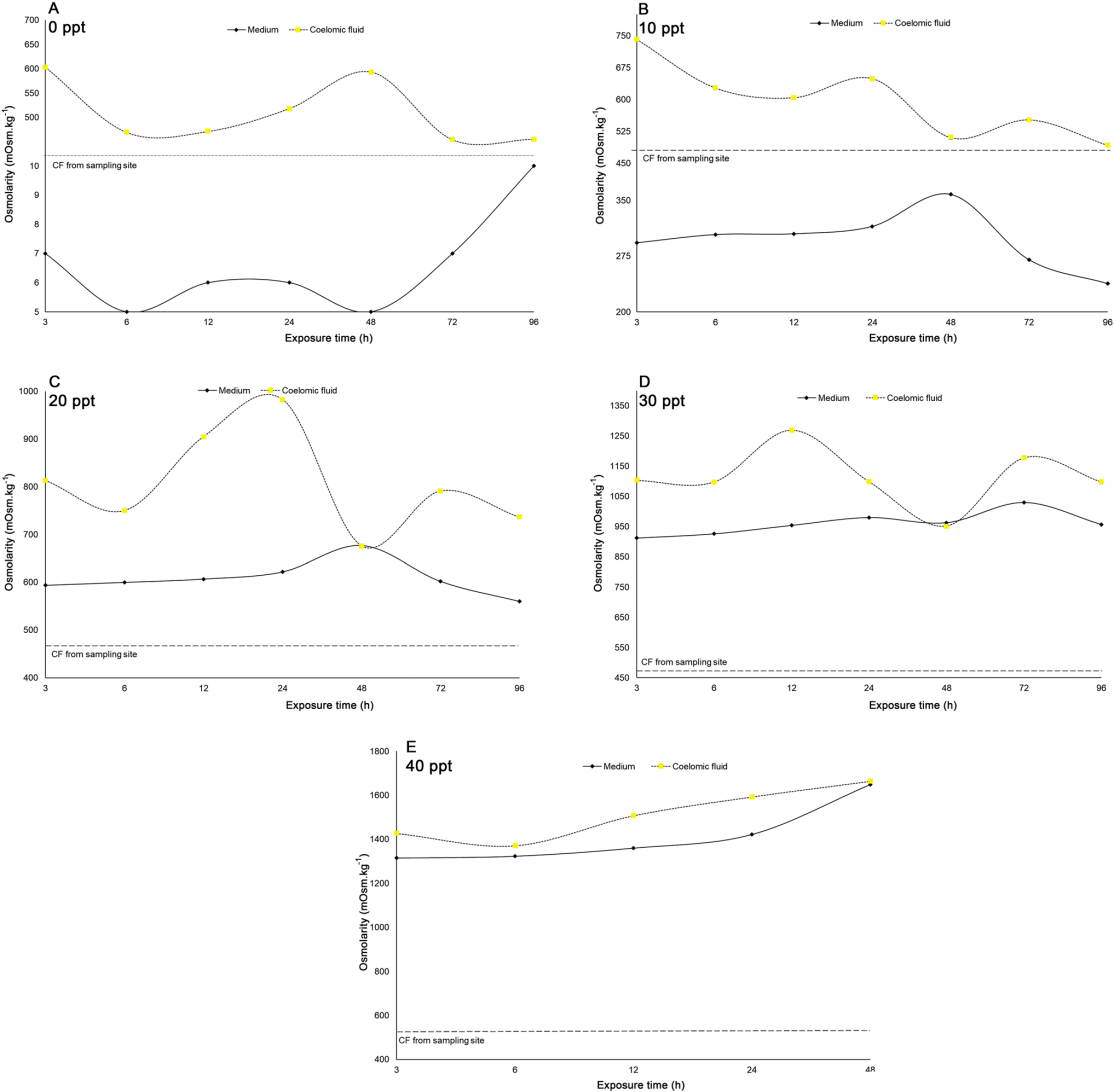
The coelomic fluid osmolarity of *P. longissimus* after exposure to different salinity concentrations of (**A**) 0 ppt, (**B**) 10 ppt, (**C**) 20 ppt, (**D**) 30 ppt, and (**E**) 40 ppt. Horizontal constant dash lines represent field values.

Field collected *P. litoralis* earthworm (Fig. 5) exhibited a coelomic fluid osmolarity of 765 mOsm kg^−1^. When they were placed into 0 ppt (1–9 mOsm kg^−1^, mean = 4 mOsm kg^−1^) test medium, they regulated the water content of the body and so although the coelomic fluid osmolarity increased, it remained hypertonic (553 mOsm kg^−1^) to the medium. A similar trend was observed when the worms were introduced into 10 ppt salinity (233–376 mOsm kg^−1^, mean = 314 mOsm kg^−1^), where the coelomic fluid osmolarity increased to 653 mOsm kg^−1^. Further increasing in the medium’s salinity of 20 ppt (533–817 mOsm kg^−1^, mean = 641 mOsm kg^−1^) and 30 ppt (783–1198 mOsm kg^−1^, mean = 961 mOsm kg^−1^), the coelomic fluid of *P. litoralis* rises until it meets isotonicity (852 mOsm kg^−1^ and 1341 mOsm kg^−1^ respectively) at 96 h of exposure. Further increased the salinity of the test medium to 40 (1309–1825 mOsm kg^−1^, mean = 1465 mOsm kg^−1^) or 50 ppt (1557–2118 mOsm kg^−1^, mean = 1748 mOsm kg^−1^) resulted in the coelomic fluid osmolarity becoming isotonic after 48 h of exposure, but the worms started to die if immersed in either of these salinities for more than 48 h. *Pontodrilus longissimus* showed a similar trend when it was placed in a 0 ppt salinity test medium (5–10 mOsm kg^−1^, mean = 6 mOsm kg^−1^), it remained hypertonic (505 mOsm kg^−1^) to the medium (Fig. 6). When they were placed into medium with a salinity of 20 ppt (560–677 mOsm kg^−1^, mean = 608 mOsm kg^−1^) or 30 ppt (912–1030 mOsm kg^−1^, mean = 960 mOsm kg^−1^), the coelomic fluid of *P. longissimus* becoming isotonic after 48 h of exposure. However, at a salinity of 40 ppt (1315–1649 mOsm kg^−1^, mean = 1413 mOsm kg^−1^), *P. longissimus* was not able to survive for more than 48 h of exposure.

## Discussion

The established cosmopolitan littoral earthworm *Pontodrilus litoralis* was found to live in various types of habitats whereas *P. longissimus* was found only in estuaries with muddy sand. Sympatry of these two *Pontodrilus* species occurred in some localities, where *P. litoralis* was repeatedly found in the sand surface, while *P. longissimus* was found at deeper levels below the surface than *P. litoralis*^6,17^. *Pontodrilus litoralis* has been reported as a bioluminescence earthworm and uses this to avoid predation by littoral predators because *P. litoralis* live on the sand surface^17–20^. We suggest that salinity is one of the key factors affecting the distribution and habitat preference of *Pontodrilus* in this region. Congeneric *P. longissimus* inhabited mostly estuaries with a lower salinity compared to *P. litoralis*.

The 28-d exposure to different salinity concentrations demonstrated a negative relationship between high salinity stress and *P. longissimus* survival, which only survived at low salinity concentrations and with a lower survival rate observed when compared to *P. litoralis*. We suggest that *P. litoralis* has a higher tolerance to salinity fluctuations than *P. longissimus* and that both species of littoral earthworms prefer to inhabit the ecotone between terrestrial and marine habitats. Our results support the current distribution patterns of these two littoral earthworm species, where *P. litoralis* is a cosmopolitan species and occurs in a very wide range of sub-temperate and tropical coastal ecosystems^4,6,7,21–24^ with dispersal mechanisms including transportation by wooden vessels^5,25^. Salinity fluctuation is a principal environmental factor affecting the distribution of marine and estuarine species^26^ and high euryhalinity may be a factor in its global distribution similar to the case of the marine intertidal gastropod *Stramonita brasiliensis*^27^. In contrast, a poor tolerance to salinity changes is a factor that limits the dispersal ability of *P. longissimus*, concordant with it only being reported in the coastline of Thailand and Peninsular Malaysia^6,8^.

The effects of salinity have also been reported in other earthworms, where it affected the quality of products in composting, limited the growth of earthworms, and reduced the weight and survival percentage of earthworms^12,28–30^. Here, the salinity was found to affect the weight change of both littoral earthworm species. The highest level of weight gain in *P. litoralis* was observed at 12 h in all salinity concentrations, which we suggest supports the notion that water enters by osmosis increasing the worm’s weight but this response becomes limited over time. The result showed that *P. litoralis* was able to resist salinity changes and match the salinity fluctuations in their natural microhabitat as previously reported in *P. bermudensis* (which is now considered a synonym of *P. litoralis*)^14^, with a salinity concentration of 20–30 ppt being the optimal range for *P. litoralis*.

Understanding the fundamental knowledge of osmotic strategies in gaining salinity tolerance should provide insights into the evolutionary processes of physiological adaptation to saline waters and habitat selection strategies. Several studies have been reported on osmoregulation in earthworms^28,31,32^. Since, osmoregulation is very important in estuarine intertidal species, we investigated how the coelomic fluid osmolality of these two *Pontodrilus* increases with increasing medium salinity to evaluate how they the species handle salinity fluctuations. Changes in salinity concentrations will cause a change in the osmotic pressure of an organism through osmoregulation processes. The body fluid osmolarity changed in accord with the outside exposure medium. These findings are in agreement with the present hypothesis and confirm that *P. litoralis* and *P. longissimus* are osmoconformers with regard to salinity change^33–35^. In conclusion, the current study examined the effects of different salinities on the two species of earthworms in the genus *Pontodrilus*. A high salinity concentration resulted in a reduced weight and survival percentage of the cosmopolitan earthworm *P. litoralis* and was fatal to the endemic *P. longissimus*. The osmoregulation ability of these two different *Pontodrilus* species of littoral earthworms to changes in salinity appear to explain their distribution pattern and habitat preference throughout Thailand’s coastal areas, where *P. litoralis* has a higher tolerance to salinity fluctuations than *P. longissimus* corresponding with its greater ability to disperse throughout the world. Habitat patterns associated with salinity indicated that their natural habits are based upon the least osmolarity stressful environment. Theoretically, a specific adaptation is required, they either control their body fluid equivalent to the composition of the sea or actively conserve or shed excess salts.

## Material and methods

### Field sampling and observation

Littoral earthworm genus *Pontodrilus* were surveyed throughout the coastal areas of Thailand, both on the Gulf of Thailand (east coast) and the Andaman Sea (west coast) from January 2015 to January 2016. The sampling sites are shown in Fig. 1. During the field collection, geographic variation between the sampling sites was observed and photographed (type of biotope), with salinity of the sampling site were recorded.

### Laboratory experiment

#### Test organism

Adult *P. litoralis* (weight 190.31 ± 30.21 mg) and *P. longissimus* (weight 532.49 ± 146.17 mg) with a well-developed clitellum were collected from Hat Bang Saen, Mueang Chonburi, Chonburi, Thailand and from Hat Pakmeng, Sikao, Trang, Thailand, respectively. Only active worms were acclimated to laboratory conditions. The earthworms were rinsed with distilled water and left for a day on wet paper, moistened with artificial seawater without soil to avoid their stomach contents.

#### Test substances and medium

The sand was transported back from the field to the laboratory and oven-dried for 24 h. Different salinities of the test medium (0, 10, 20, 30, 40 and 50 ppt) were prepared by adding artificial sea salt to distilled water, and the salinity was then adjusted accordingly using a refractometer (ATAGO, Master-S10M).

### Experimental procedure

#### Long-term (28 d) salinity exposure

A similar working procedure was applied to the long-term exposure (28 d) where the earthworms were exposed to different salinities (0, 10, 20, 30, 40 and 50 ppt) in the presence of the sand as a substrate. Round plastic containers (internal diameter 170 mm, height 130 mm) were filled with 2 kg of sand. Each container had an inlet and an outlet to allow for running water and cleaning processes and was lined with a paper bag in order to prevent the worms from escaping out of the container. Ten earthworms were released into each container with four replicates of each treatment. The tests were conducted at ambient temperature (30 ± 2 °C) and a 16:8 h light:dark cycle (light intensity of 400–800 lx). Finely ground cow dung (30 g) was added each week to each container as food^36^. At the end of the exposure, the survival rate and weight change of each treatment were calculated.

#### Short-term (0–96 h) salinity exposure

The short-term exposure test was conducted in a round, flat-bottomed (internal diameter 48 mm, height 80 mm) container. Each test container was filled with 20 ml of the prepared medium with the designated salinity level (artificial seawater at 0, 10, 20, 30, 40 and 50 ppt) and then exposed for different duration (3, 6, 12, 24, 48, 72 and 96 h). Each treatment was performed with three replicates (one individual per replicate). The test units were closed with a perforated plastic film to prevent the earthworms from escaping. The experiment was carried out at ambient room temperature (25 ± 2 °C) with a 16:8 h light:dark illumination cycle (light intensity of 400–800 lx). The working medium was checked and changed every day in order to avoid salinity changes due to evaporation. The behavior of the earthworms was recorded using a Canon 650D camera. The earthworms were weighed after 0, 3, 6, 12, 24, 48, 72 and 96 h of exposure, and the change in weight was calculated. The earthworms were prepared for osmolality testing.

#### Data analysis

One-way ANOVA was used to evaluate differences among different treatments on the assessed parameters, while Tukey’s post hoc test was performed to test for the significance of any differences between the means of different treatments. All statistical analyses were performed using the Minitab 16 Statistical software and significance was accepted at P < 0.05 level.

#### Determination of the body (coelomic) fluid osmolarity

Earthworms from the short-term exposure experiment were used for osmolality determination. Their coelomic fluid (≥ 20 μL) was extracted from the coelomic cavity with the aid of a thin glass capillary. A Micro Osmometer (Fiske Model 210 Micro Osmometer) was used to determine the osmolality of each solution using freezing point depression and was calibrated using the manufacturer-supplied standards.

## Supplementary Information

Supplementary Information.

## Data Availability

The data that support the findings of this study are openly available in Zenodo at https://doi.org/10.5281/zenodo.7214872.
