# Supplementary material for: Responses to salinity in the littoral earthworm genus Pontodrilus
Source: Sci Rep. 2022 Dec 24;12:22304. doi: 10.1038/s41598-022-26099-w (PMC9789941; doi:10.1038/s41598-022-26099-w)
Supplement: Supplementary file 1 — Supplementary Information. [file 41598_2022_26099_MOESM1_ESM.pdf]

Supplementary Materials

Responses to salinity in the littoral earthworm genus *Pontodrilus*

Teerapong Seesamut[1], Beewah Ng[1, 2], Chirasak Sutcharit[1], Ratmanee Chanabun[3], Somsak Panha[1,4]*

[1]Animal Systematics Research Unit, Department of Biology, Faculty of Science, Chulalongkorn University, 254 Phayathai Road, Pathumwan, Bangkok 10330, Thailand
[2] Freecap Resource Sdn Bhd, Lot T-5, Lumut Port Industrial Park, KG Acheh Mukim Lumut, 32000 Sitiawan, Perak, Malaysia
[3] Program in Animal Science, Faculty of Agricultural Technology, Sakon Nakhon Rajabhat University, Sakon Nakhon 47000, Thailand
[4]Academy of Science, The Royal Society of Thailand, Bangkok 10300, Thailand

Supplementary Materials legends

Supplementary Table 1. Observed localities of the two *Pontodrilus* species in this study, showing salinity records and habitat characteristics.

Supplementary Figure 1. Photographs showing habitats of *Pontodrilus litoralis* and *P. longissimus*. (**A**) Sanitary sewer emptying to the sandy beach, (**B**) mangrove swamp, (**C** and **D**) under the trash or leaf litter on the sandy beach, and (**E** and **F**) estuaries.

Supplementary Figure 2. Size comparison of *Pontodrilus litoralis* after exposure to salinity at (**A**) 0 ppt and (**B**) 50 ppt (photographed after a 12 h exposure).

Supplementary Table 1. Observed localities of the two *Pontodrilus* species in this study, showing the salinity records and habitat characteristics.

Abbr.	Species	Observation locality	Date	Salinity	Habitat
A1	*P. litoralis*	Koh Chang, Koh Chang, Trat	16 Dec 2015	30	Under the trash or leaf litter on the sandy beach
A2	*P. litoralis*	Ao Dong Tai, Sattahip, Chonburi	18 Dec 2015	29	Mangrove swamp
A3	*P. litoralis*	Hat Cha-am, Cha-am, Phetchaburi	15 Jan 2016	1	Sanitary sewer emptying to the sandy beach
A4	*P. litoralis*	Laem Sai, Chaiya, Surat Thani	14 Jan 2016	9	Sanitary sewer emptying to the sandy beach
A5	*P. litoralis*	Hat Sai Kaew, Singha Nakhon, Songkhla	13 Jan 2016	2	Mangrove swamp
A6	*P. litoralis*	Ao Thalen, Mueang, Krabi	11 Jan 2016	33	Mangrove swamp
B1	*P. longissimus*	Hat Bang Sak, Takua Pa, Phangnga	5 Dec 2015	4	Estuary
B2	*P. longissimus*	Nang Thong Bay Resort, Takua Pa, Phangnga	5 Dec 2015	1	Estuary
B3	*P. longissimus*	Hat Mai Khao, Talang, Phuket	5 Dec 2015	3	Estuary
AB1	*P. litoralis* *P. longissimus*	Hat Sai Ngoen, Klong Yai, Trat	15 Dec 2015	14	Estuary
AB2	*P. litoralis* *P. longissimus*	Hat Chao Lao, Thamai, Chanthaburi	8 Aug 2015	1	Estuary
AB3	*P. litoralis* *P. longissimus*	Klong Bang Siap, Patiew, Chumphon	4 Aug 2015	19	Estuary
AB4	*P. litoralis* *P. longissimus*	Hat Koey, Kaper, Ranong	10 Jan 2016	7	Estuary
AB5	*P. litoralis* *P. longissimus*	Hat Pak Meng, Sikao, Trang, Thailand	30 Aug 2015	20	Estuary
AB6	*P. litoralis* *P. longissimus*	Hat Bo Chet Look, La Ngu, Satun	12 Jan 2016	21	Estuary

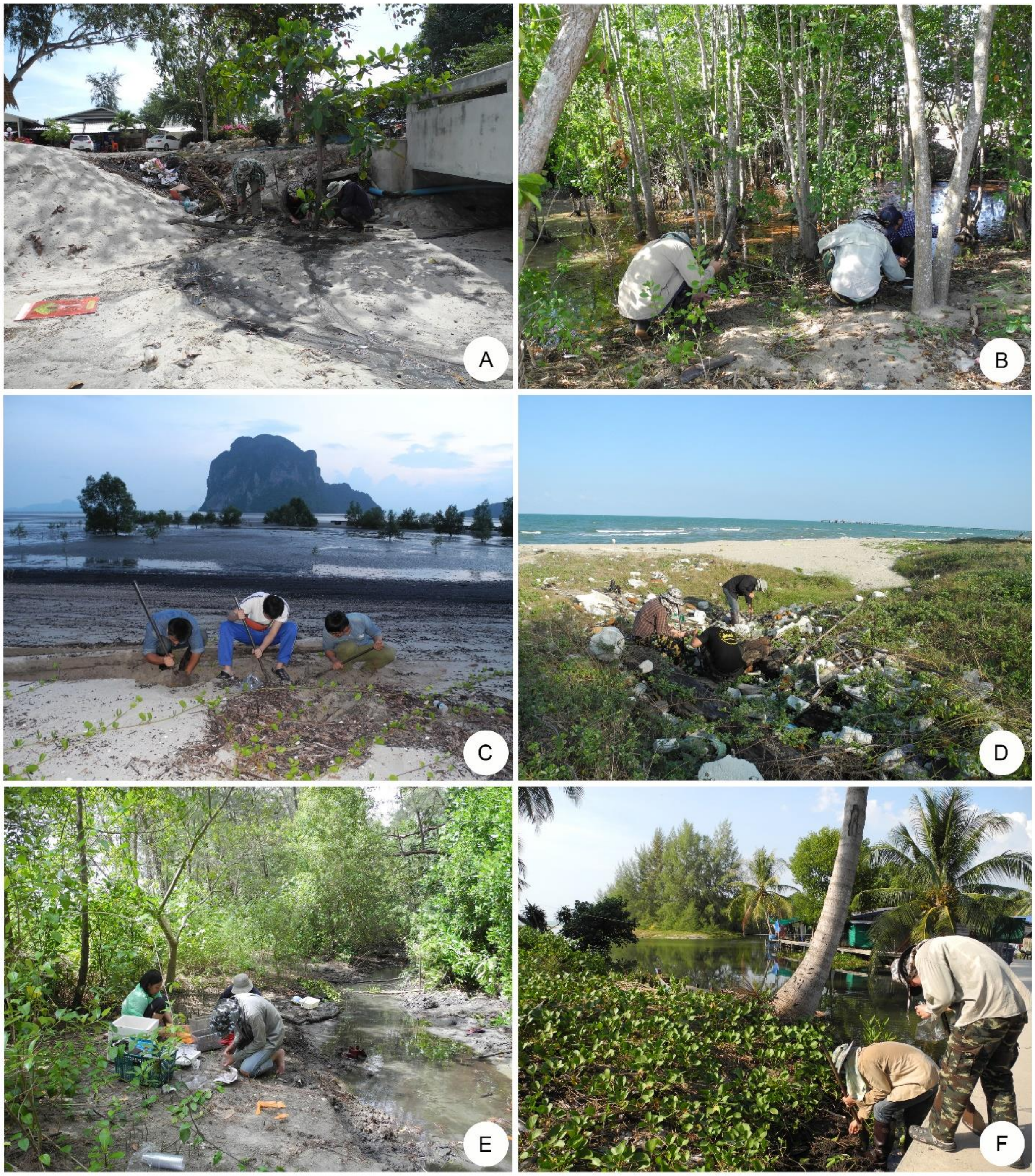

Supplementary Figure 1. Photographs showing habitats of *Pontodrilus litoralis* and *P. longissimus*. (**A**) Sanitary sewer emptying to the sandy beach, (**B**) mangrove swamp, (**C** and **D**) under the trash or leaf litter on the sandy beach, and (**E** and **F**) estuaries.

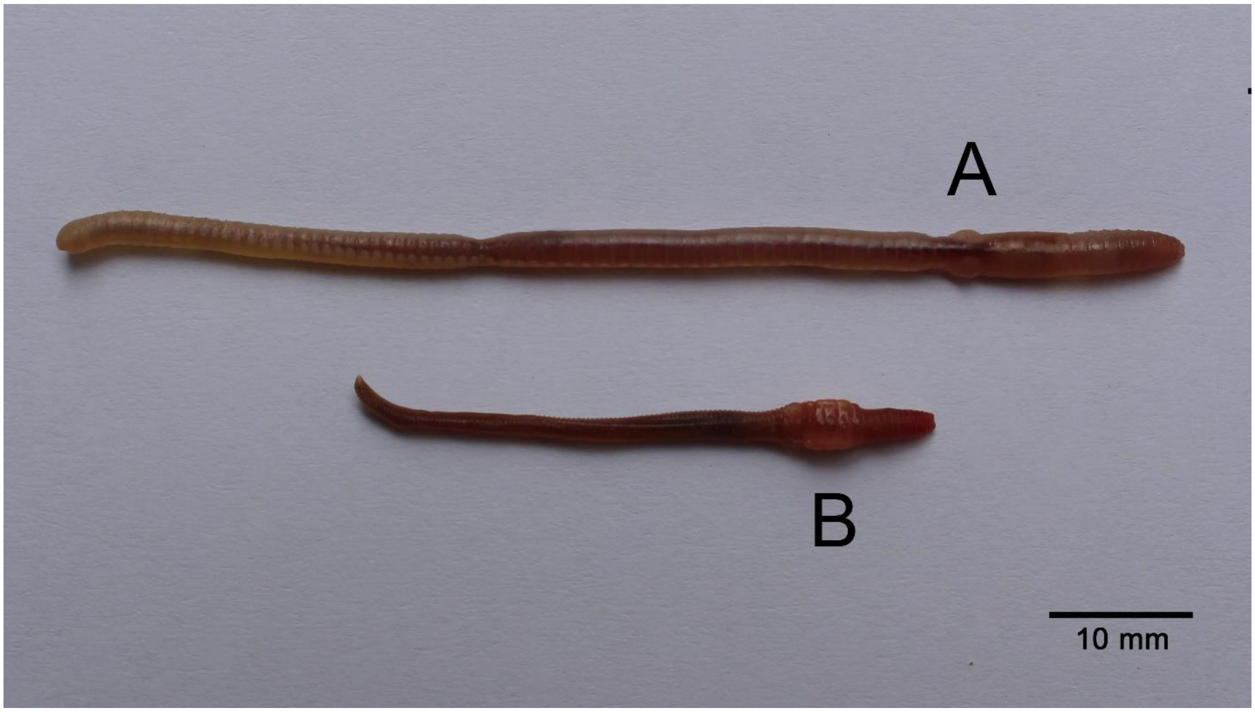

Supplementary Figure 2. Size comparison of *Pontodrilus litoralis* after exposure to salinity at (**A**) 0 ppt and (**B**) 50 ppt (photographed after a 12 h exposure).
